# The Role of Alterations in Alpha-Klotho and FGF-23 in Kidney Transplantation and Kidney Donation

**DOI:** 10.3389/fmed.2022.803016

**Published:** 2022-05-06

**Authors:** Meera Gupta, Gabriel Orozco, Madhumati Rao, Roberto Gedaly, Hartmut H. Malluche, Javier A. Neyra

**Affiliations:** ^1^Department of Surgery - Transplant Division, University of Kentucky, College of Medicine, Lexington, KY, United States; ^2^Department of Surgery, University of Kentucky, Lexington, KY, United States; ^3^Department of Internal Medicine - Nephrology, Bone and Mineral Metabolism Division, University of Kentucky, College of Medicine, Lexington, KY, United States; ^4^Division of Nephrology, Department of Medicine, University of Alabama at Birmingham, Birmingham, AL, United States

**Keywords:** alpha-Klotho, FGF-23, kidney donation, kidney transplant, ESKD (end stage kidney disease), bone mineral density (BMD), cardiovascular disease, mineral bone disease

## Abstract

Cardiovascular disease and mineral bone disorders are major contributors to morbidity and mortality among patients with chronic kidney disease and often persist after renal transplantation. Ongoing hormonal imbalances after kidney transplant (KT) are associated with loss of graft function and poor outcomes. Fibroblast growth factor 23 (FGF-23) and its co-receptor, α-Klotho, are key factors in the underlying mechanisms that integrate accelerated atherosclerosis, vascular calcification, mineral disorders, and osteodystrophy. On the other hand, kidney donation is also associated with endocrine and metabolic adaptations that include transient increases in circulating FGF-23 and decreases in α-Klotho levels. However, the long-term impact of these alterations and their clinical relevance have not yet been determined. This manuscript aims to review and summarize current data on the role of FGF-23 and α-Klotho in the endocrine response to KT and living kidney donation, and importantly, underscore specific areas of research that may enhance diagnostics and therapeutics in the growing population of KT recipients and kidney donors.

## Introduction

Cardiovascular disease (CVD) and bone mineral disorders are important causes of mortality and morbidity among end-stage kidney disease (ESKD) patients (1). The endocrine adaptation to a decreased glomerular filtration rate (GFR) plays a central role in the cardiovascular and skeletal alterations observed in patients with chronic kidney disease—mineral bone disorder (CKD-MBD). Fibroblast growth factor 23 (FGF-23) and its coreceptor, α-Klotho, are key factors in phosphate homeostasis, and their dysregulation is an essential link between osteodystrophy, left ventricular hypertrophy, atherosclerosis, systemic inflammation, and renal fibrosis observed in patients with ESKD (2).

While the treatment of choice for ESKD is kidney transplant (KT), CVD continues to be a significant cause of morbidity and mortality after transplantation and is a contributing factor to graft failure and loss (3). Post kidney transplant patients (PKTP) have persistent alterations in bone mineral metabolism exacerbated by immunosuppressive therapy, which can lead to progression of bone disease and CVD (4). While the bone-vascular axis has been extensively studied and is significantly affected in patients with ESKD, the endocrine response to kidney transplantation and kidney donation is not yet fully understood. Among kidney donors, significant derangements of markers of bone metabolism, including serum α-Klotho, have been reported after nephrectomy (5–9). However, the clinical impact, if any, of these alterations in otherwise healthy living donors has not been determined.

In this manuscript, we aim to review and summarize current data on the role of FGF-23 and α-Klotho in the endocrine response to KT and living kidney donation. Importantly, we intend to underpin specific areas of research that may enhance diagnostics and therapeutics in the growing population of KT recipients and kidney donors.

## FGF-23 and α-Klotho in Mineral Metabolism

FGF-23 is one of the few fibroblast growth factors that enters the systemic circulation and acts as a hormone (10). FGF-23 is synthesized by osteocytes and secreted in response to rising serum phosphate levels. Its secretion is also stimulated by parathyroid hormone (PTH), vitamin D, soluble α-Klotho, and pro-inflammatory cytokines including IL-1, IL-6, and TNFα (2, 11–13). All fibroblast growth factor receptors (FGF-R) have a low affinity for FGF-23, and the high-affinity interaction depends on the expression of the co-receptor, α-Klotho (14, 15).

**Figure 1:**
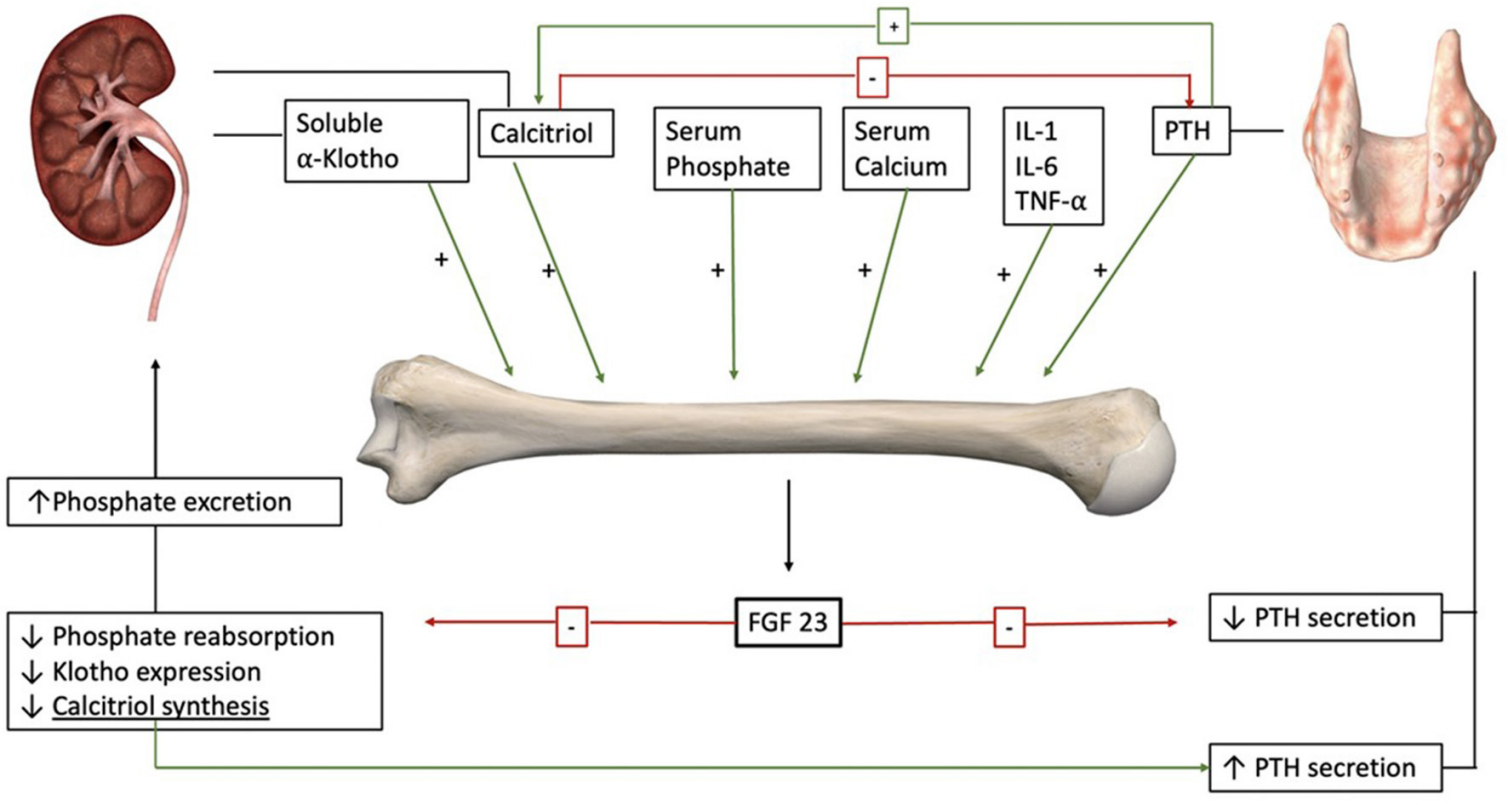
FGF-23 is secreted by osteocytes in response to different endocrine and mineral stimuli including PTH and calcitriol. The main targets of FGF-23 are the kidneys and the parathyroid glands. In the kidneys, it increases phosphate excretion and decreases calcitriol synthesis. Decreased levels of calcitriol increase PTH secretion. On the parathyroids glands, the direct effect of FGF-23 is inhibitory. Also, FGF-23 downregulates the expression of its co-receptor α-Klotho (anatomical images courtesy of Essential Anatomy ^@^3D4Medical).

The organs most impacted by FGF-23 are the kidneys and parathyroid glands (Figure 1). In the absence of disease, FGF-23 induces the degradation and decline in the synthesis of sodium-dependent phosphate transport protein 2A (NaPi2A) in the kidney (16). In doing so, FGF-23 directly decreases proximal tubular reabsorption of phosphate (17). Highlighting its physiological relevance, animal models with a deletion on FGF-23 or α-Klotho genes develop phosphate retention (18). FGF-23 also modifies mineral metabolism by decreasing calcitriol levels through two different mechanisms: it decreases calcitriol synthesis by downregulating 25-hydroxyvitamin D_3_ 1α hydroxylase in the proximal tubular cells of the kidney, and it increases vitamin D inactivation by up-regulating vitamin D 24-hydroxylase (CYP24A1) expression (19, 20). The decrease in serum calcitriol contributes to PTH secretion despite a direct inhibition of the parathyroid glands by FGF-23 (20, 21). FGF-23 also promotes calcium absorption in the distal convoluted tubules, counterbalancing the inhibition of calcitriol synthesis (22). Notably, FGF-23 auto-regulates its effects by inhibiting the expression of α-Klotho (23). Hence, FGF-23 has a significant direct and indirect effect on phosphate and calcium metabolism.

α-Klotho, discovered as a gene linked to aging, is predominantly expressed in the kidney, specifically in the distal tubules (24, 25). The α-Klotho gene encodes a 130 kDa single-pass transmembrane protein consisting of two extracellular domains (KL1 and KL2), a transmembrane domain, and a short cytoplasmic tail. Part of the extracellular domain of transmembrane α-Klotho is cleaved by proteases (26–28). A secreted form of α-Klotho has also been postulated from an alternatively spliced transcript (29, 30). Cleaved α-Klotho proteins are released into the circulation and are referred to as soluble α-Klotho. Soluble α-Klotho acts as an endocrine or paracrine factor affecting multiple distant organs such as the heart, lungs, bone, and brain (31–37). α-Klotho increases FGF-23 expression in the bone (36) and acts as a non-enzymatic scaffold protein that enhances FGF-23 signaling (38).

α-Klotho is an important regulator of mineral metabolism homeostasis: (1) it decreases renal phosphate reabsorption by acting as a co-receptor for FGF-23 binding to FGFR1 (15); (2) it directly promotes the internalization and degradation of the NaPi2a cotransporter in the renal proximal tubules (39); and (3) it suppresses vitamin D signaling (α-Klotho knockout mice can be rescued from a phenotype of soft tissue calcification by deletion of the CYP27B1 gene) (40–42). In addition, α-Klotho has a plethora of pleiotropic actions as an inhibitor of apoptosis, fibrosis, and cell senescence (43–46).

## FGF-23 and α-Klotho in Chronic Kidney Disease and Its Complications

Patients with CKD have significantly reduced functional renal cell mass, leaving the residual renal parenchyma to maintain homeostasis by increasing phosphate excretion. FGF-23—through its phosphaturic effects— plays a crucial role in this response (2, 17) and is an early biomarker of CKD (47). As intact FGF-23 is cleared by the kidneys, a decreased glomerular filtration rate (GFR) also independently contributes to its rising levels (48–50). Among patients with CKD, sustained elevated levels of FGF-23 lead to constant inhibition in calcitriol synthesis, with a consequent increase in PTH secretion (51, 52). Because PTH stimulates the release of FGF-23 synthesis, a closed loop of stimulation is created. Supraphysiologic levels of FGF-23 are also perpetuated by downregulation of its co-receptor α-Klotho, resistance to its renal action, and further hyperphosphatemia (53–56). Remarkably, the compensation to impaired kidney function might promote further progression of CKD through different mechanisms: the phosphate hyperfiltration by functional nephrons leads to tubular damage (57), downregulation of α-Klotho promotes renal fibrosis through increasing TGFβ1 (44) and Wnt (58) signaling pathways, and lower α-Klotho levels might decrease its protective effect on the glomerular filtration barrier (59). The resulting endocrine profile that characterizes patients with CKD –high FGF-23 and PTH, with low calcitriol and α-Klotho—contributes to cardiovascular morbidity, systemic inflammation, dysregulation of bone metabolism, and anemia.

### Cardiovascular Diseases

Cardiovascular diseases cause more than half of all deaths among patients with ESKD, and they represent the leading cause of mortality in this vulnerable population (1). Among CKD patients older than 65 years, the prevalence of CVD is 65.8%, compared to 31.9% in patients of the same age group but with normal kidney function (60). Epidemiological studies have shown a direct association between FGF-23 levels, mortality, and risk of major cardiovascular events (61–63). Two major contributor mechanisms are left ventricular hypertrophy (LVH) and a pro-atherosclerotic milieu. LVH is associated with heart failure, changes in heart geometry, and cardiac arrhythmias, while atherosclerosis is associated with coronary, cerebrovascular, and peripheral artery diseases.

High circulating levels of FGF-23 directly induce LVH through the activation of FGFR4 in cardiac myocytes and recruitment of the PLCγ/Calcineurin/NFAT pathway independently of α- Klotho (64–66). FGFR4 is a potential therapeutic target for reducing cardiovascular risk in patients with CKD as pharmacological blockage of this receptor in animal models showed attenuated LVH, while loss of function (FGFR4^−/−^) protected mice from developing LVH (64).

The pro-atherosclerotic milieu characteristic of patients with CKD is related to alterations in the bone-kidney axis (60, 67). In the initial stages of CKD, increased secretion of FGF-23 helps maintain normal serum phosphate (47). As the GFR decreases, transient or continuous hyperphosphatemia leads to calcium phosphate precipitation and favors the formation of atherosclerotic plaques (68). There is a positive correlation between FGF-23 and vascular calcification in patients with CKD; however, it is still controversial if the association is independent of serum phosphate concentrations or a direct pro-atherosclerotic effect of FGF23 (69).

Aortic stiffness is another biomarker of cardiovascular risk that increases in patients with CKD. While atherosclerosis primarily affects the vascular intima, aortic stiffness represents degenerative changes in the extracellular matrix of the intima and media layers characterized by elastin fracture and collagen deposition (70). Smith et al. demonstrated that increased aortic stiffness among CKD patients is associated with elevated circulating calciprotein particles—which are formed by adsorption of calcium phosphate by Fetuin-A to prevent ectopic calcification (71, 72). Also, *in-vitro* studies have shown that hyperphosphatemia can trigger chronic inflammation, induce endothelial apoptosis and promote an osteoblastic phenotype in vascular smooth muscle cells (2, 73–75). These are other examples of how mineral metabolism affects the vascular system and increases cardiovascular risk in CKD.

### Immune System and Inflammation

The systemic inflammation observed in patients with CKD contributes to their elevated morbidity and mortality (76–78). An association between renal function and inflammation is well-established, and eGFR is inversely correlated with circulating levels of pro-inflammatory cytokines (e.g., IL-1β, IL-6, TNF-α, and hs-CRP) (79). Factors that promote inflammation in CKD include increased production of cytokines, hyperphosphatemia, chronic and recurrent infections, acidosis and oxidative stress, altered metabolism of adipose tissue, and gut microbiota dysbiosis (80). FGF-23 directly induces liver secretion of inflammatory cytokines by targeting FGFR4 receptors in hepatocytes and activating PLCγ/Calcineurin/NFAT signaling pathway— independently of α-klotho. As an indicator of the therapeutic potential of this finding, blockage of FGFR4 prevented significant elevation in C-Reactive Protein (CRP) secretion by the liver in an animal model of CKD (76). Also, altered immune homeostasis increases the susceptibility to infections. FGF-23 impairs neutrophil recruitment by interfering with chemokine signaling through activation of FGFR2 (81). In an unfavorable feedback loop, FGF-23 increases hepatic secretion of cytokines, inflammatory cytokines stimulate the secretion of FGF-23 by osteocytes, and a predisposition to infections contributes to systemic inflammation.

### Bone Metabolism

There is a wide range of bone disorders that affect patients with CKD. The spectrum includes disorders in bone turnover, impaired mineralization, and changes in bone volume (82, 83). These abnormalities cause decreased mechanical strength with increased risk of fractures (84, 85), and altered mineral metabolism with increased risk of ectopic calcification (86). Malluche et al. reported a series of 630 bone biopsies from patients with CKD on dialysis. With 418 cortical bone biopsies available, they found that 82% had altered bone turnover, 52% had high cortical porosity, cortical thickness was low in 44%, cancellous bone volume was altered in 69% of the cohort, and only 3% of patients had impaired mineralization. Notably, bone turnover was low among 58% of patients and high among 24% of patients (87). These data show the high prevalence and variety of bone alterations in patients with CKD. High bone turnover is related to elevated PTH, hyperphosphatemia, and increased cortical porosity. Conversely, low bone turnover is related to low calcium retention and ectopic calcification. Low calcium retention indicates impaired incorporation of calcium into the bone matrix with the consequent hypercalcemia after calcium loads (88).

Animal models have shown that FGF-23 also regulates bone mineralization through a phosphate-independent mechanism. Homozygous FGF-23 knock-out mice develop growth delay, low bone mineral density (BMD), skeletal malformations, increased bone fragility, and impaired osteoid mineralization with concomitant hyperphostemia (89). Furthermore, these skeletal abnormalities persisted in hypophosphatemic animal models (FGF-23 ^−/−^ / NaPi2a ^−/−^) (90). On the other hand, in fetal rat calvaria cell cultures overexpressing FGF-23, there was also inhibition of bone mineralization (91). This apparent contradiction—where deficiency and exposure to FGF-23 lead to impaired bone mineralization—favors the possibility that FGF-23 acts as an autocrine/paracrine regulator of mineralization (92) and the inhibition of tissue-non-specific alkaline phosphatase activity (TNAP) by FGF-23 might play a role. TNAP hydrolyzes pyrophosphate and generates inorganic phosphate to enhance mineralization (93). FGF-23 can suppress TNAP activity, cause pyrophosphate accumulation, and inhibit mineralization. The deletion of FGF-23 in Hyp mice (a murine homolog for X-linked hypophosphatemia) rescued the suppressed TNAP activity in osteoblasts (93, 94). However, FGF-23 deficiency (*Fgf*^−/−^ mice) increases TNAP activity, increases the release of osteopontin, and also leads to inhibition of mineralization. This inhibitory effect of osteopontin in bone mineralization can be attenuated with a low phosphate diet or by deletion of the NaPi2A transporter (92, 95).

Osteocytes and osteoblast synthesize α-Klotho (96). Mice with an osteocyte-specific deletion of the α-Klotho gene have increased osteoblast activity and increased bone volume with normal serum biochemistry. When the same deletion is introduced in mice models of CKD (5/6 nephrectomy and high-phosphate diet)—which present with elevated serum phosphate, PTH, and FGF-23—α-Klotho expression in the bone was significantly decreased and the pro-osteoblastic effect was no longer significant. This suggests that α-Klotho might act as a regulator of adequate bone mineralization rate when the systemic mineral homeostasis is preserved (92, 97). As described, the endocrine mechanisms affecting bone metabolism are complex and questions remain as to the role of FGF-23 and α-Klotho in the differential expression of bone disorders observed in patients with CKD.

### Anemia

Epidemiological studies have found that FGF-23 is independently associated with anemia (98, 99), and animal studies have reported possible causal mechanisms. The administration of FGF-23 in wild-type mice rapidly decreases erythropoiesis while Knockout mice (FGF-23 ^−/−^) have increased circulating erythropoietin and red blood cell number (100). Furthermore, Agoro et al. reported that inhibiting FGF-23 signaling rescues anemia and iron deficiency in a mice model of CKD by normalizing the hemoglobin levels, decreasing erythroid cell apoptosis, and increasing serum erythropoietin, iron, and ferritin. They also found an up-regulation on the liver synthesis of hepcidin that normalized after FGF-23 blocking and provided a possible link between FGF-23, inflammation, and anemia (101).

In summary, elevated serum FGF-23 is an early biomarker of impaired renal function that directly contributes to the morbidity observed in patients with CKD. Clinical and animal studies have shown that potential therapeutic benefits of targeting FGF-23 include attenuating left ventricular hypertrophy, decreasing the synthesis of pro-inflammatory cytokines by the liver, improving neutrophil recruitment, rescuing anemia by stimulating erythropoiesis, and improving iron deficiency by down-regulating the synthesis of hepcidin. Decreased circulating levels of α-klotho is also characteristic of patients with CKD and potential therapeutic benefits of targeting α-klotho-dependent actions include increasing circulating levels of calcitriol, decreasing renal fibrosis, and improving bone mineralization and osteoblast function.

## Post Kidney Transplant Patients

Kidney transplantation is associated with a significant reduction in mortality compared to those with ESKD on dialysis. Wolfe et al. reported a 1.7-fold decrease in the annual death rate among patients with ESKD undergoing transplant compared to patients on waiting list (102). Notably, CVD persists as the leading cause of death after transplantation (60). According to the USRDS, the prevalence of CVD is 76.5% in patients on hemodialysis, 65% in patients on peritoneal dialysis, and 53.7% in patients with a functional kidney transplant (1). Life expectancy among kidney transplant recipients remains inferior compared to age-matched controls from the general population. For example, females who undergo kidney transplantation between 40 and 44 years old have a projected lifespan of more than 10 years shorter than similarly aged women from the general population (1). For this reason, it is paramount to identify and characterize the mechanisms underlying CVD in kidney transplant recipients.

Transplant mineral and bone disorder (T-MBD) is a systemic condition characterized by endocrine alterations, vascular or other soft-tissue calcification, and abnormalities in bone metabolism (103, 104). Although there are significant differences with CKD-MBD, there is a continuum in some metabolic pathways conducive to atherosclerosis and bone disorders (104). There are several mechanisms involving the aforementioned biomarkers after kidney transplant with persistent interplay affecting mineral bone metabolism, cardiovascular disease, and load-bearing capacity.

After transplant, as renal function stabilizes, there is a tendency toward normalization of most biochemical indicators of ESKD. A recovered GFR allows excretion of excess serum phosphate and uremic toxins. High basal levels of PTH and FGF-23 make post-transplant hypophosphatemia and hypercalcemia a common finding (105–110). Transient hypophosphatemia is observed in more than 80% of transplant recipients early after surgery, and is related to appropriate allograft function and favorable outcomes (106). On the other hand, transient hypercalcemia is reported in up to 65% of transplants and is associated with poor outcomes (111–113). Hypercalcemia can compromise adequate graft function by acutely inducing vasoconstriction and, in the long-term, by favoring calcinosis and vascular calcification (114, 115).

In the first 3 months following surgery, there is a significant decrease in PTH, FGF-23, and calcium levels and an increase in serum phosphate and calcitriol toward normal levels (107). After this period, these changes normalize at a slower rate. Nonetheless, long-term endocrine alterations are common (106, 107, 109, 116). Among 50 patients undergoing kidney transplantation, Evenepoel et al. reported that 1 year after surgery, 57% had persistently high FGF-23, and 32% had high serum PTH levels. Mild to moderate hypophosphatemia was also present in 14% of patients (117). Elevated FGF-23 is associated with decreased post-transplant GFR (118, 119) and, and increased risk of mortality, graft loss (120, 121), and left ventricular wall strain (119, 121). The effects of FGF-23 in the cardiovascular system were also studied by Yilmaz et al. who reported that endothelium-dependent vasodilation improves after kidney transplantation, and it is independently associated with a decrease in FGF-23 and serum phosphorus (122). As mentioned above, high circulating levels of FGF-23 directly contribute to left ventricular hypertrophy (LVH) and anemia in patients with CKD. Decreases in FGF-23 might contribute to the reduction of LVH and improvements of ventricular contractility after kidney transplantation (123, 124). Baloglu et al. reported that hemoglobin levels are inversely associated with circulating FGF-23, however this association was no longer significant after kidney transplantation (125).

Serum α-Klotho levels increase after transplantation and might contribute to the transient hypophosphatemia by improving the sensitivity to FGF-23 (126, 127). Interestingly, Mizusaki et al. reported that serum α-Klotho was significantly higher in kidney transplant recipients receiving everolimus. They concluded that mTOR inhibition could be related to increased α-Klotho after kidney tranplantation (127), which underpins a new area of applicable α-Klotho therapeutics in kidney transplantation that needs further study. Higher serum α-Klotho has also been independently associated with improved GFR 1 year after transplant (128). Animal models have shown a reno-protective effect of α-Klotho that could play a role in protecting the renal allograft (129–131). Sugiura et al. reported that α-Klotho induction through gene transferring strategy mitigates apoptosis, histological damage and improves GFR in mice subject to renal ischemia-reperfusion injury. They concluded that the protective mechanism involved increases expression of heat shock protein 70 (129). The antifibrotic effect of α-Klotho through the inhibition of TGFβ1 and Wnt signaling pathways is also an important mechanism in kidney protection against injury (44, 58). Hu et al. studied mice after acute kidney injury and found that early treatment with α-Klotho prevents progression to CKD and significantly reduces uremic cardiomyopathy (37). As an indicator of renal function, α-Klotho activity varies depending on the quality of the transplanted kidney. Among kidneys from deceased donors, lower Kidney Donor Risk Index (KDRI) and age <50 years were associated with higher α-Klotho levels and better GFR after transplant (128, 132). In this context, α-Klotho represents a potentially useful biomarker and therapeutic agent in kidney transplantation.

Bone metabolism and load-bearing capacity are also greatly affected in kidney transplant recipients. The underlying mechanism is multifactorial and includes pre-existing renal osteodystrophy, reduced kidney function, and the effect of immunosuppressive therapy (133, 134). Among 68,814 kidney transplant recipients, Nikkel et al. reported a 22.5% incidence of fractures during the first 5 years after surgery (135). The first 6 months following transplant are associated with a rapid decrease in spine bone mineral density that continues to decline afterward but at a slower rate (136–138). It is well-established that immunosuppressive therapy with glucocorticoids induces osteopenia and is a contributing factor to osteodystrophy. Glucocorticoids are related to a decreased number and impaired function of osteoblasts. They also promote osteoclastic activity by up-regulating RANKL expression and down-regulating osteoprotegerin (139–141). Conversely, the effect of calcineurin inhibitors in the bone is more controversial and difficult to evaluate (141–143). While tacrolimus and cyclosporine have been linked to osteoporosis, population-based studies have failed to find an association with an increased risk of fractures (141, 143–146). Briner et al. conducted a clinical study that combined cyclosporine treatment with no or very low dose of glucocorticoids. They found that glucocorticoid-sparing immunosuppression after transplant prevented cancellous bone loss, and they did not observe any fractures during the 2 years follow-up (147). Also, Westeel et al. reported a series of 52 renal transplanted patients receiving low dose prednisone and cyclosporine. They concluded that cyclosporine, together with the decrease of prednisone dosage, contributes to a transient stimulation of bone remodeling at 6 months after transplant, which counterbalances the bone loss associated with prednisone therapy (142). In addition to immunosuppressive treatment, post-transplant hypophosphatemia is also independently associated with alterations in bone turnover, decreased osteoblast activity, defective mineralization, and osteoblast apoptosis (133, 148). In this regard, high PTH and FGF-23 levels are frequent after transplant and contribute to hypophosphatemia. Persistent tertiary hyperparathyroidism also leads to increased bone turnover, and high FGF-23 has been related to phosphate-independent skeletal abnormalities (89, 90, 149). Human studies of soluble α-Klotho and FGF-23 among kidney transplant recipients are summarized in Table 1.

**Table 1:** Human studies of soluble α-Klotho and FGF-23 among kidney transplant recipients.

**Study**	**Clinical setting**	**Methods**	**Observations**	**FGF-23 and α-Klotho highlights**
Evenepoel et al. (117)	50 KTRs and 50 eGFR-matched controls followed for one year after transplant.	FGF-23 (Serum ELISA)	- FGF-23 levels tend to normalize after successful renal transplant. - Elevated FGF-23 levels (>50 ng/L) were observed in 66% and 57% at month 3 and month 12, respectively. - At month 3, FGF-23 levels were significantly higher than controls. Statistical significance was lost after one year. - Hyperphosphatoninism and renal phosphorus wasting also regressed by 1 year after transplantation.	FGF-23 tends to normalize after renal transplantation.
Trombetti et al. (105)	69 KTRs patients followed evaluated 10-13 days after transplant.	FGF-23 (Serum ELISA)	- Higher serum FGF-23 levels were independently associated with lower serum phosphate and decreased renal tubular reabsorption of phosphate after transplant. - FGF-23 was inversely associated with serum calcitriol levels and directly associated with serum calcium levels. - Serum levels of PTH and FGF-23 were not correlated.	Preoperative serum FGF-23 is associated with post-transplant phosphate, calcium, and calcitriol levels.
Kawarazaki et al. (108)	39 KTRs were evaluated one week before and 12 months after transplant.	FGF-23 (Serum ELISA)	- Pre-transplant FGF-23 was the best pre-transplant predictor of persistent hypophosphatemia one year after kidney transplant.	Preoperative FGF-23 is a predictor of persistent post-transplant hypophosphatemia.
Wolf et al. (120)	984 KTR were followed up for three years.	FGF-23 (Serum ELISA)	- During a median follow-up of 37 months, 87 patients died and 101 patients had allograft loss. - High levels of FGF-23 were independently associated with all-cause mortality and graft loss.	FGF-23 levels are associated with all-cause mortality and graft loss in stable kidney transplant recipients.
Han et al. (107)	20 KTRs followed for 12 weeks.	FGF-23 (Serum ELISA)	- FGF-23 levels decreased by 97% at 4 weeks after transplant but were still above normal. - FGF-23 levels, but not PTH levels, were independently associated with post-transplant hypophosphatemia.	Preoperative FGF-23 levels are associated with post-transplant hypophosphatemia.
Sanchez et al. (118)	Cross-sectional study of 279 maintenance kidney recipients with CKD stages 1-4 and stable allograft function.	FGF-23 (Serum ELISA)	- FGF23, PTH, and phosphorus levels were higher in more advanced stages of CKD, while serum calcitriol levels and phosphate reabsorption rate were lower. - A significant inverse correlation was found between eGFR and FGF23. - FGF-23 was inversely correlated with serum phosphate levels. - High FGF-23 was independently associated with increased time on corticosteroids, increased PTH, increased serum phosphate, and decreased calcitriol. - Higher FGF23 values were not correlated with increased phosphate excretion.	Serum FGF-23 levels remain elevated after renal transplantation and are inversely correlated with eGFR.
Wesseling-Perry et al. (109)	44 pediatric KTRs followed for 6 months after transplant.	FGF-23 (Urinary and serum ELISA)	- Higher pre-transplant FGF-23 values were associated with an increased fractional excretion of phosphorus and a faster decline in circulating phosphate during the first week after transplantation. - Circulating 1,25(OH)2 vitamin D levels increased more rapidly and were consistently higher in patients with lower FGF23 values. - 25(OH) vitamin D and 24,25(OH)2 vitamin D values were unrelated to FGF23 concentrations. - Post-transplantation fractional excretion of FGF-23 was less than 5% at all post-operative times, even in the patients with highest circulating FGF-23	Higher pre-transplant FGF-23 is related with faster decline in serum phosphate and slower increase in calcitriol levels after renal transplantation.
Baia et al. (121)	593 stable KTRs were evaluated 2.7-11.7 years after transplantation and followed for 6.2 - 7.5 years.	FGF-23 (Serum ELISA)	- FGF23 was independently associated with markers of left ventricular wall strain (pro-A-type natriuretic peptide, N-terminal-pro brain natriuretic peptide, and copeptin). - FGF23 serum level was associated with a higher risk of cardiovascular and all-cause mortality.	Among long-term kidney transplant recipients, higher FGF-23 is associated with markers of left ventricular wall strain, cardiovascular mortality, and all-cause mortality.
Yilmaz et al. (122)	161 adult KTRs were evaluated before and 6 months after transplant.	FGF-23 (Serum ELISA)	Endothelium-dependent vasodilatation improved after kidney transplantation, and it was independently associated with a decrease in FGF23 and serum phosphorus, and an increase in 25-OH-VitaminD levels.	FGF-23 is associated with improved endothelium-dependent vasodilation after kidney transplantation.
Malyszko et al. (119)	84 KTRs with stable graft function and no clinical signs of rejection. Control group: healthy volunteers.	α-Klotho and FGF-23 (Serum ELISA)	- FGF23 was significantly higher and Klotho significantly lower in kidney transplant recipients compared with healthy volunteers. - FGF-23 significantly correlated with IL-6, VCAM, and copeptin. α-Klotho significantly correlated with N-Terminal pro brain natriuretic peptide: a marker of left ventricular wall strain. - FGF23 was significantly higher and Klotho significantly lower in patients with eGFR >60 mL/min compared with patients with eGFR <60 mL/min. - They concluded that disturbances in the FGF23-Klotho system appeared to be related to the endothelial cell injury	Among kidney transplant recipients, there is an association between markers of endothelial cell injury with high FGF-23 and low α-klotho levels.
Bleskestad et al. (110)	Cross-sectional study of 39 adults KTRs with more 10 years after first transplant. 39 eGFR matched controls. 20 healthy controls.	α-Klotho and FGF-23 (Serum ELISA)	- Among KTR, FGF23 and PTH were significantly higher than in healthy volunteers. - When compared with matched controls for eGFR, PTH was significantly higher and levels of FGF23 had a non-significant trend towards higher levels and α-Klotho towards lower levels.	Among long-term kidney transplant recipients there are persistently high levels of FGF-23 and PTH.
Tartaglione et al. (116)	Cross sectional study of 80 adult KTRs.	30 healthy controls α-Klotho and FGF-23 (Serum ELISA)	- Serum level of FGF23 was significantly increased in KTR. - Serum α-Klotho was significantly decreased in KTR. - FGF-23 had a direct correlation with sclerostin levels (bone-antianabolic protein), - α-Klotho had a significant positive association with sclerostin levels on multivariate analysis.	Sclerostin – a bone antianabolic protein– has a positive association with FGF-23 and α-Klotho levels in kidney transplant recipients.
Tan et al. (126)	29 KTRs were prospectively evaluated before transplant and at 1, 12 and 52 weeks after transplant.	α-Klotho and FGF-23 (Serum ELISA)	- α-Klotho was significantly increased at 52 weeks following an initial decline at 1 week. - FGF23 was significantly lower at 52 weeks.	FGF-23 decreases, and α-Klotho increases after renal transplantation.
Mizusaki et al. (127)	36 KTRs were evaluated before and 1 year after transplantation.	α-Klotho (Serum ELISA)	- α-Klotho levels were higher after transplantation than before transplantation. - After transplant, α-Klotho levels were significantly higher in recipients taking everolimus than in those not taking everolimus. - mTOR inhibition may augment the increase in Klotho levels in transplant recipients.	α-Klotho levels were significantly higher in renal transplant recipients receiving immunosuppressive treatment with mTOR inhibitors (everolimus).
Thongprayoon et al. (9)	431 KTRs; meta-analysis with variable follow-up	α-Klotho (Serum ELISA)	-KTRs had a significant increase in serum klotho levels at 4 to 13 months post-KT. - KT recipients had a lower serum klotho level compared to healthy unmatched volunteers.	Serum α-Klotho increases after kidney transplantation, but it is still lower than healthy volunteers.

*CKDs, chronic kidney disease patients; ELISA, enzyme-linked immunoassay; FGF, fibroblast growth factor; eGFR, estimated glomerular filtration rate; KTRs, kidney transplant recipients; KT, Kidney transplantation*.

## Kidney Donors

In 2020, living kidney donors represented 23% of the total transplanted kidney allografts in the US. The long-term mortality after kidney donation has proven to be similar to the general population or matched controls in several studies (150–152). However, the innocuity of kidney donation is the subject of greater debate. O'Keeffe et al. conducted a meta-analysis that included 118,426 living kidney donors with an average follow-up of one to 24 years. They did not find evidence of increased mortality, cardiovascular diseases, or diabetes among donors compared to controls (153). Nonetheless, they found that donors had an increased risk of ESKD and pre-eclampsia. Muzaale et al. conducted a cohort study involving 96,217 kidney donors and found that 15 years after donation, the risk of ESKD increased from 0.04% in matched non-donors to 0.31% in kidney donors (154). There are scarce data available evaluating the outcomes after donation beyond a follow-up of 20 years. Based on OPTN data as of 2020, 26.5% of kidney donors were under 35 years old. In this regard, an increasing population is expected to live longer than 40 years after donation (155), and the long-term metabolic consequences of unilateral nephrectomy in otherwise healthy individuals are not clearly understood.

As discussed above, α-Klotho is primarily synthesized in the kidneys, and it has a reno-protective effect through different mechanisms. Shortly after kidney donation, α-Klotho levels decrease and remain lower than baseline 1 year after surgery (5–8, 156). However, one study that evaluated serum α-Klotho 5 years after donation did not find a significant difference compared to healthy controls (157). The long-term consequences of changes in α-Klotho levels in response to nephrectomy remain unknown. Further, the scope of the possible therapeutic benefit of increasing α-Klotho levels to prevent the development of ESKD after nephrectomy has not been explored in humans.

Other parameters of the bone-kidney axis are also altered after donation. There is a decrease in GFR, phosphate, and calcitriol synthesis, and an increase in PTH that persists 1 year after surgery (5). However, there is conflicting information about the changes in FGF-23 after donation. Some authors have reported that FGF-23 remains elevated 1 year after donation (6, 158), while others did not find significant long-term changes (5, 157, 159). A meta-analysis conducted by Thongprayoon et al. reported no significant change in FGF-23 1 year after kidney donation. Notably, they also found that serum α-Klotho remained lower than baseline 1 year after nephrectomy among 56 donors (9). As such, there is limited available data considering the clinical relevance of alterations in FGF-23 and α-Klotho after kidney donation (159). Human studies of soluble α-Klotho and FGF-23 among kidney donors are summarized in Table 2.

**Table 2:** Human studies of soluble α-Klotho and FGF-23 among living donors.

**Study**	**Clinical setting**	**Methods**	**Results/Observations**	**FGF-23 and α-Klotho highlights**
Westerberg et al. (159)	9 LKDs followed for 6 months post-donation	Serum ELISA	- FGF-23 levels were negatively correlated with serum phosphate and calcitriol prior to nephrectomy - FGF-23 levels increased from 31.8 ±12.3 pg/mL to 55.8 ±15.1 pg/mL one week after nephrectomy, and did not correlate with other measures, and return to baseline at 3 months - FGF-23 correlated with PTH at 3 and 6 months	FGF-23 increased after nephrectomy and returned to baseline by 3 months after surgery.
Young et al. (158)	198 LKDs and 98 non-donor controls followed 5.3 years post-donation	Serum Urine ELISA	- Median time after donation was 5.3 years - FGF-23 was higher (38.9 vs 29.7 pg/mL) and eGFR was lower in donors compared to controls - Donors had higher urine phosphate and iPTH, but lower serum calcitriol and phosphate levels - iPTH did not correlate with FGF-23 or calcitriol - Unclear if changes impact MBD or fracture rates	Long-term follow up after nephrectomy showed persistently high FGF-23 and decreased eGFR among kidney donors.
Akimoto et al. (7)	10 LKDs and their LKRs followed for 5 days post-donation/transplant	Serum ELISA	- Baseline serum α-Klotho was 909.8 pg/mL (IQR 754.8−1132.4) among LKDs and 613.0 pg/mL (IQR 445.9-750.8) among recipients prior to surgery - Postoperative day 5, serum α-Klotho among donors dropped to 619 pg/mL (IQR 560.3−811.8 pg/mL), while no change in α-Klotho levels was observed in kidney recipients	After kidney donation, serum α-Klotho decreased by post-operative day five when compared to pre-operative levels.
Ponte et al. (5)	27 LKDs followed for 12 months post-donation	Serum Urine IBL ELISA	-FGF-23 and α-Klotho drop within days post kidney donation - 12 months post-donation, LKDs have lower eGFR and vitamin D3, unchanged vitamin D2, higher PTH, lower serum phosphate, unchanged FGF-23, and slightly improved but still lower α-Klotho levels compared to pre-donation	One year after kidney donation, serum α-Klotho and eGFR were lower and FGF-23 is similar to pre-operative levels.
Kimura et al. (8)	15 LKDs and 6 LKRs followed for 5 days post-donation/transplant	Serum Urine IBL ELISA	- Baseline serum α-Klotho was 827.9 pg/mL (IQR 544.7−993.3) among recipients and 1084 pg/mL (IQR 794.8-1638.2) among LKDs prior to surgery - Postoperative days 2 and 5, serum α-Klotho among donors dropped by −23.9 and −25.3 pg/mL, respectively; serum α-Klotho levels among recipients also decreased by −25.7 and −15.5 pg/mL, respectively - Baseline urine α-Klotho was 58.6 ng/day (IQR 29.3−142.1) among recipients and 698.8 ng/day (62.3−1619.5) among LKDs - Urinary α-Klotho increased well above baseline on postoperative days 2 and 5 among both groups suggesting that the kidney is a major source of urinary α-Klotho	After kidney donation, serum α-Klotho decreased and urinary α-Klotho increased by postoperative day five when compared to pre-operative levels.
Thorsen et al. (157)	35 LKDs 15 years (median) post-donation, 60 CKDs, and 35 controls	Serum Plasma ELISA	- FGF-23 levels among LKDs [62.6 pg/mL(IQR 6.6−112)] were no different than controls [51.8 pg/mL(25.9−90.0)], but significantly lower than levels among advanced ESKDs - α-Klotho levels among LKDs [669.3 pg/mL(IQR 409−1161)] were no different than controls [725.4 pg/mL(IQR 458−1222), but significantly lower among CKDs - NGAL was higher among LKDs than controls (2.02 ± 0.10 vs 1.89 ± 0.10 pg/mL; p <0.001), but much lower compared to CKDs - FGF-23 and α-Klotho levels are not significantly different between LKDs long-term after donation and controls	Long-term, FGF-23 and α-Klotho levels were similar between living kidney donors and healthy controls.
Tan et al. (6)	21 LKDs and 20 controls followed for 12 months post-donation	Serum IBL ELISA	- Biochemical and clinical profiles among LKDs and controls were similar pre-donation - One month post-donation, serum creatinine increased (from 75 ± 12 to 114 ± 22 μmol/L), FGF23 increased (52 ± 15 to 70 ± 19 pg/mL) and α-Klotho decreased (564 [469-662] to 424 [375-523] pg/mL), all P less than 0.001. - Changes persisted 12 months post-donation, while there were no alterations in serum phosphate, PTH, urinary phosphate among LKDs.	One years after kidney donation, FGF-23 was higher and α-Klotho lower when compared to healthy controls.
Thongprayoon et al. (9)	108 LKDs and 431 KTRs; meta-analysis with variable follow-up	Serum ELISA	- LKDs had a decrease in α-Klotho (mean −232.24 pg/mL) 3−5 days post-donation, and slight recovery but remained below baseline one year post-donation (mean −110.80 pg/mL); and lower than healthy volunteers (mean −92.41 pg/mL) at one year post-donation - There was no difference in FGF-23 (mean +8.19 pg/mL) among LKDs one year post-donation - KTRs had increase in α-Klotho within the first year post-transplant (mean +243.11 pg/mL), comparable to eGFR-matched controls	One year after kidney donation, serum α-Klotho was lower when compared to pre-operative levels and healthy volunteers. FGF-23 was not similar to pre-operative levels.
Hiemstra et al. (156)	36 LKDs and 36 controls followed for 12 months	Serum IBL ELISA	- α-Klotho among LKDs declined early post-donation compared to controls [677.7pg/mL(536.7−833.9)vs 893.4pg/mL(739.8−1051.0)], but remained significantly lower than controls at 6-weeks [701.6 pg/mL(548.6−874.0)] and 12 months [721.4 pg/mL(562.5−956.5)] - eGFR among LKDs declined markedly early post-donation compared to controls (52.3 ± 17.5 vs 83.6 ± 25.2), but remained lower at 6-weeks (60 ± 20 vs 87.2 ± 25.9) and 12 months (67.7 ± 22.6 vs 88.6 ± 24.3) - There was no difference in FGF-23 between LKDs and controls at all-time points	Serum α-Klotho and eFGR decreased early post kidney donation and remained low one year after surgery when compared to healthy controls. There was no difference in FGF-23 between LKDs and controls at all-time points

*LKDs, living kidney donors; ELISA, enzyme-linked immunoassay; FGF, fibroblast growth factor; iPTH, intact parathyroid hormone; MBD, mineral bone disease; IQR, interquartile range; eGFR, estimated glomerular filtration rate; KTRs, kidney transplant recipients; CKDs, chronic kidney disease patients*.

The understanding of long-term adaptations to kidney donation is still the subject of intense investigation. Living kidney donors are essential to close the gap between supply and kidney allograft demand, and sustaining their long-term health is important. Further research and education are necessary to support the kidney health of kidney donors and ensure the maintenance of their endocrine, cardiovascular, and bone mineral health.

## Conclusion

FGF-23 and α-Klotho are integral endocrine factors that link bone-mineral metabolism, kidney function, and cardiovascular health. Among patients with CKD, this multi-looped axis is significantly impaired and translates into greater cardiovascular morbidity, mortality, and bone disorders. Kidney transplantation partially restores α-Klotho activity and FGF-23 levels. However, persistently high FGF-23 is associated with increased mortality and graft loss, and low circulating α-Klotho is associated with decreased kidney function after transplant. Notably, the metabolic bone and vascular alterations observed post kidney transplant are not fixed and instead are dynamic, influenced by organ quality, kidney recovery, immunosuppression, recipient comorbidities, and associated treatments. This highlights the concept that patients continue to need personalized medical support after transplant to ensure normalization of endocrine and bone-vascular axes indirectly affected by CKD. On the other hand, while some authors have reported that kidney donors have lower α-Klotho levels and elevated FGF-23 that persist 1 year after surgery, the scarce available data are inconclusive regarding the clinical relevance of these findings.

## Author Contributions

MG, GO, and JN contributed to conception and design of the study. MG and GO wrote the first draft of the manuscript. JN wrote sections of the manuscript. All authors contributed to manuscript revision, read, and approved the submitted version.

## Conflict of Interest

The authors declare that the research was conducted in the absence of any commercial or financial relationships that could be construed as a potential conflict of interest.

## Publisher's Note

All claims expressed in this article are solely those of the authors and do not necessarily represent those of their affiliated organizations, or those of the publisher, the editors and the reviewers. Any product that may be evaluated in this article, or claim that may be made by its manufacturer, is not guaranteed or endorsed by the publisher.
